# Frequency of Human Immunodeficiency Virus in Patients Admitted with Acute Stroke

**DOI:** 10.7759/cureus.8296

**Published:** 2020-05-26

**Authors:** Rahmat Ali, Muhammad Inam Khan, Muhammad Omer Sultan, Umar Farooque, Syed Adeel Hassan, Fahham Asghar, Omer Cheema, Sundas Karimi, Choudhary Ahmed Hasan, FNU Farukhuddin

**Affiliations:** 1 Internal Medicine, United Medical and Dental College, Karachi, PAK; 2 Internal Medicine, Jinnah Postgraduate Medical Center, Karachi, PAK; 3 Neurology, Dow Medical College, Dow University of Health Sciences, Karachi, PAK; 4 Neurology, Dow University of Health Sciences, Karachi, PAK; 5 Internal Medicine, Dow University of Health Sciences, Karachi, PAK; 6 General Surgery, Combined Military Hospital, Karachi, PAK; 7 Neurology, University Hospital Cleveland Medical Center, Cleveland, USA

**Keywords:** seropositive, stroke, cd4 count, frequency, dyslipidemia, male, hiv

## Abstract

Introduction

Stroke is a leading cause of chronic disability and death in both developing and developed countries. A significant proportion of stroke patients are human immunodeficiency virus (HIV) positive. About half of the HIV patients experience some sort of neurological deficit in their lifetimes. The exact reason for the occurrence of stroke in HIV infected patients is poorly understood. The purpose of our study is to determine the frequency of HIV among patients admitted with acute stroke.

Materials and methods

This cross-sectional study is conducted at a major metropolitan hospital in Karachi for six months. A total of 130 patients of stroke between the ages of 30 and 70 years of either gender were included in this study. A complete history was taken and a physical examination was performed. Each patient underwent a battery of tests that included HIV serology, lipid profile, electrocardiography (ECG), chest X-ray (posteroanterior (PA) view), and computed tomography (CT) scan of the brain. Carotid Doppler ultrasonography to assess carotid artery stenosis was also ordered. The means and standard deviations of age and cluster of differentiation 4 (CD4) cell count were calculated. The frequencies and proportions of gender, diabetes mellitus (DM), hypertension (HTN), smoking, obesity, dyslipidemia, carotid artery stenosis, and HIV status were calculated. Stratification was done by applying the chi-square test and assuming p-value ≤0.05 as significant. This helped analyze the association of age, gender, DM, HTN, smoking, obesity, dyslipidemia, and carotid artery stenosis to the frequency of HIV.

Results

The mean age of the study population was 55.54 ± 11.166 years. There were 39 (30%) patients <50 years of age while 91 (70%) patients were ≥50 years of age. Gender distribution showed that 86 (66.15%) patients were male, and 44 (33.85%) patients were female. Furthermore, 71 (54.62%) patients were hypertensive, 53 (40.77%) were diabetic, 62 (47.69%) were smokers, 49 (37.69%) were obese, 52 (40%) had dyslipidemia, and 77 (59.23%) had carotid artery stenosis. The frequency of HIV was noted at 24 (18.46%). The mean CD4 count was estimated at 241 ± 103.295 cells/mm^3^. Stratification showed a significant relationship between the frequency of HIV with only gender (p=0.01) and dyslipidemia (p=0.037).

Conclusion

HIV infection in patients with stroke is not uncommon. Patients who are male, younger in age, have dyslipidemia, belong to a low socioeconomic class, or have a bad sexual history are more likely to have HIV as an underlying cause of stroke. The exact pathogenesis of such a stroke and the role of antiretroviral therapy in the prevention and treatment of this group of stroke are not completely understood and need further analysis.

## Introduction

Stroke is an important cause of chronic physical disability and mortality. The risk of its occurrence increases with age. Overall, the five-year risk of recurrent stroke is estimated at 20% [1]. According to the World Health Organization (WHO), it will continue to be the second leading cause of death in 2020.

A recent study estimated that the prevalence of stroke and/or transient ischemic attack is 21.8% in Karachi, Pakistan [2]. In Pakistan, the mortality rate of stroke is estimated to be between 7% and 20% [3].

There are many risk factors of stroke, and the human immunodeficiency virus (HIV) infection adds to that list [4-5]. According to the study conducted by Mlay et al., the prevalence of HIV is 20.9% among stroke patients [6]. About 50% of HIV patients present with cerebral infarction or transient neurological deficits in their lifetimes [7]. The exact underlying mechanism remains unclear. The hypothesized pathogenesis includes the formation of a cardiac embolus due to the development of HIV-related dilated cardiomyopathy. Furthermore, it is thought that the HIV-associated elevated systemic inflammation and the toxic effects of viral antigen leading to vascular damage and atherosclerosis can be the underlying mechanisms [8-11]. According to another study, the carotid intima-media thickness in HIV patients was more than that in non-HIV patients of the same age. The thickness advanced more quickly than earlier stated rates in non‐HIV cohorts and was related to the vascular risk factors and a low cluster of differentiation 4 (CD4) count of ≤200 cells/mm^3^. This proposes that immunodeficiency and traditional vascular risk factors can predispose to atherosclerosis [12].

The incidence of HIV and acquired immunodeficiency syndrome (AIDS) is increasing in Pakistan. Therefore, the present study is designed to determine the frequency of HIV in patients admitted with acute stroke.

## Materials and methods

Study design and sampling

This cross-sectional study took place at Jinnah Postgraduate Medical Center, Karachi, from March 20, 2019, to September 20, 2019 (six months). The non-probability consecutive sampling technique was used. The sample size was calculated by taking the prevalence of HIV in stroke patients as 20.9%, confidence interval at 95%, and margin of error at 7%. The input of the aforementioned information in Epi Info 7 (Centers for Disease Control and Prevention, Atlanta, Georgia) estimated the sample size of 130 patients. Inclusion criteria were age 30-70 years, either gender, and a confirmed diagnosis of stroke. Exclusion criteria included a space-occupying lesion on a computed tomography (CT) scan of the brain, signs of meningeal irritation, such as neck stiffness or a positive Kernig test, and a history of other serious comorbidities such as heart failure (ejection fraction (EF) < 40 on echocardiography), chronic obstructive pulmonary disease (forced expiratory volume in one second (FEV1) < 70% predicted on pulmonary function testing (PFT)), or malignancy (with documented proof).

Data collection

Stroke patients meeting the inclusion criteria were selected for the study. All participants were educated about the study before inclusion and informed consent was taken. A detailed history and thorough physical examination were carried out in all patients. Each patient underwent a list of investigations, including HIV serology, lipid profile, electrocardiography (ECG), chest X-ray (posteroanterior (PA) view), and CT scan of the brain. During hospitalization, the patients were subjected to carotid Doppler ultrasonography to assess the degree of carotid artery stenosis. Patients with HIV positive tests were recorded in the proforma by the researcher. Patients were provided with routine medical care during the hospitalization.

Data analysis

Data were entered and analyzed on SPSS version 19 (SPSS Statistics, Chicago, IL). Mean and standard deviation was calculated for continuous variables such as age and CD4 cell count. Frequency and percentage were calculated for categorical variables such as gender, diabetes mellitus (DM), hypertension (HTN), smoking, obesity, dyslipidemia, carotid artery stenosis, and the patient outcome variable, i.e. the frequency of HIV. Stratification of age, gender, DM, HTN, smoking, obesity, dyslipidemia, and carotid artery stenosis was done, the chi-square test was applied, and p-value ≤0.05 was considered significant.

## Results

The mean ± standard deviation age of the patients was 55.54 ± 11.166 years, as shown in Table 1.

**Table 1:** Analysis of age

	Minimum	Maximum	Mean	Standard Deviation
Age	39	70	55.54	11.166

**Figure 1:**
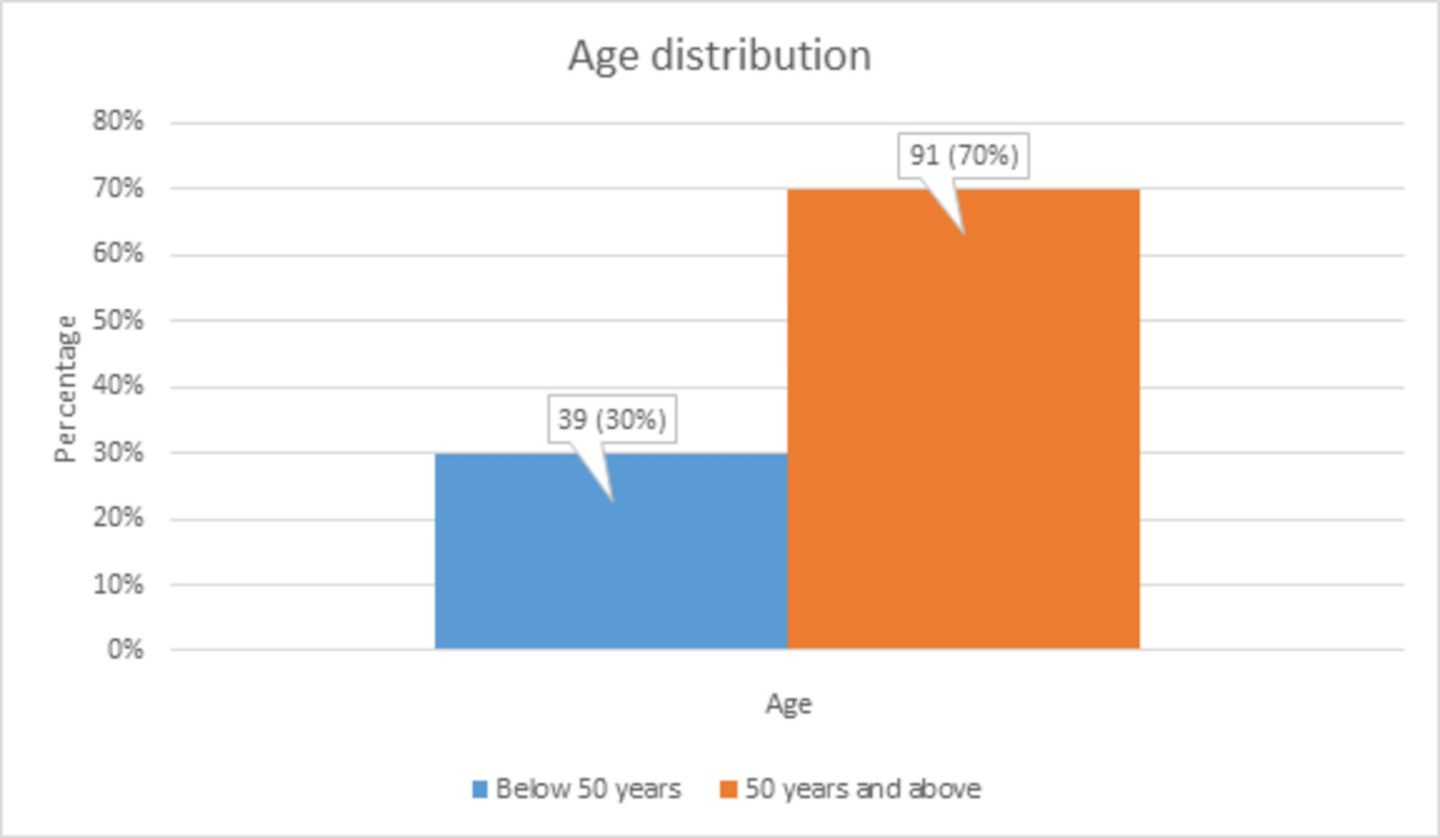
Age distribution

There were 39 (30%) patients <50 years of age and 91 (70%) patients ≥50 years of age, as shown in Figure 1.

**Figure 2:**
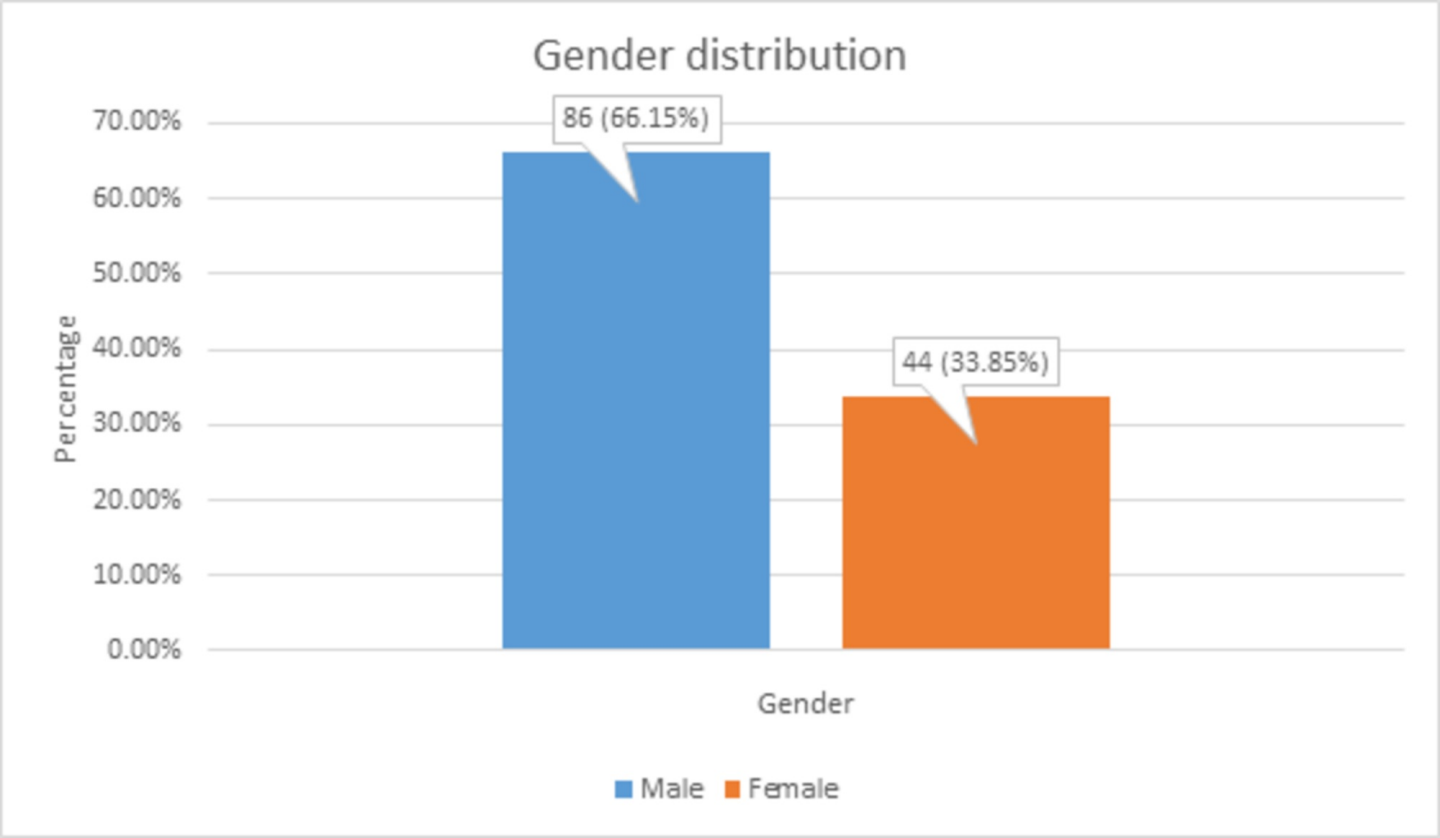
Gender distribution

There were 86 (66.15%) male patients and 44 (33.85%) female patients, as shown in Figure 2.

**Figure 3:**
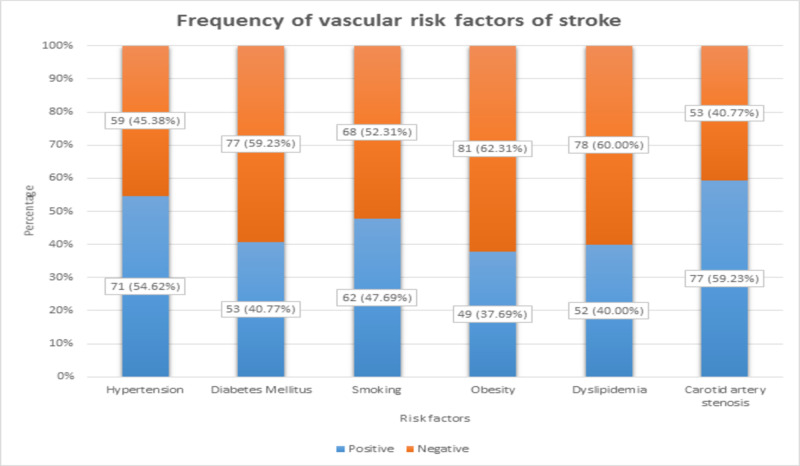
Frequency of the vascular risk factors of stroke

There were 71 (54.62%) patients with HTN, 53 (40.77%) patients with DM, 62 (47.69%) patients with positive smoking status, 49 (37.69%) patients with obesity, 52 (40%) patients with dyslipidemia, and 77 (59.23%) patients with carotid artery stenosis. These frequencies of vascular risk factors of stroke are shown in Figure 3.

**Figure 4:**
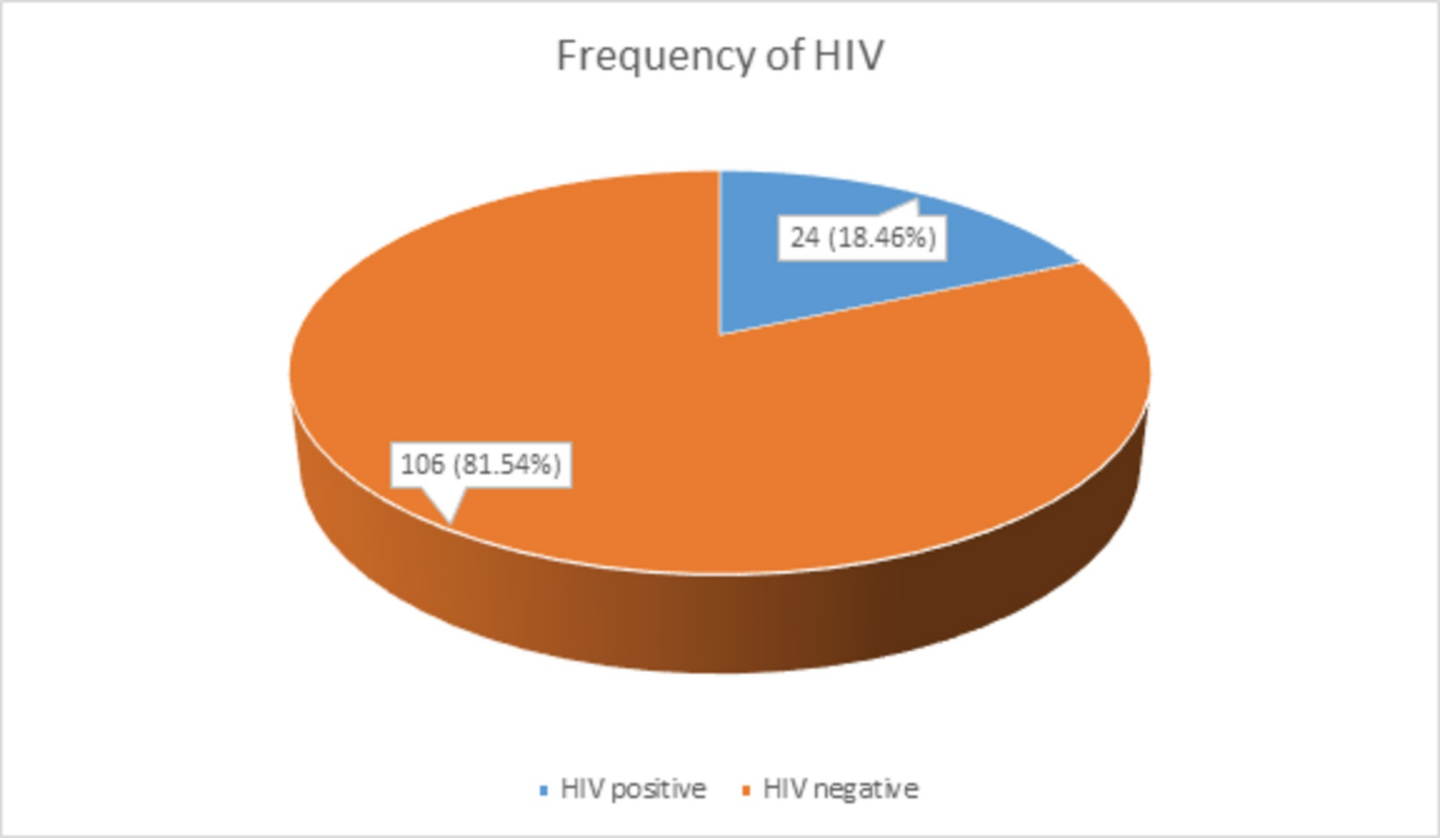
Frequency of HIV HIV: human immunodeficiency virus

On analysis of the frequency of outcome variable, 24 (18.46%) patients were HIV positive, as shown in Figure 4. The mean ± standard deviation CD4 count was 241 ± 103.2955 cells/mm^3^.

Stratification of age, gender, HTN, DM, smoking, obesity, dyslipidemia, and carotid artery stenosis with HIV prevalence indicated a statistically significant relationship only in gender (p=0.01) and dyslipidemia (p=0.037), as shown in Table 2.

**Table 2:** Stratification of demographic variables and vascular risk factors of stroke with HIV prevalence HIV: human immunodeficiency virus

Parameters		HIV positive		p-value
		Yes	No	
Age	Below 50 years	11	28	0.061
	50 years and above	13	78	
Gender	Male	21	65	0.010
	Female	03	41	
Hypertension	Yes	13	58	0.569
	No	11	48	
Diabetes Mellitus	Yes	12	41	0.214
	No	12	65	
Smoking	Yes	11	51	0.211
	No	13	55	
Obesity	Yes	11	38	0.247
	No	13	68	
Dyslipidemia	Yes	14	38	0.037
	No	10	68	
Carotid artery stenosis	Yes	11	66	0.106
	No	13	40	

## Discussion

This study estimates the frequency of HIV infection among patients with acute stroke at 18.5%, which has a statistically significant relationship with male gender and dyslipidemia. Recognition of HIV as a cause of stroke may lead to the early initiation of antiretroviral therapy. Hence, the results of this study warrant better medical management of this group of stroke patients.

In the past decade, stroke incidence has raised by 100% in developing countries [13]. This staggering rise is mainly because of the increased incidence of vascular risk factors, increased proportions of the old population, and infections such as HIV [14]. As HIV infection is most prevalent in developing countries, the chance of one patient having both stroke and HIV coincidentally is high. Although HIV infection is a risk factor for stroke, its treatment may further increase the risk because it makes HIV patients able to live longer and get continuously exposed to a suppressed HIV infection and because of its possible role in vascular damage [15-17].

The most important risk factors for stroke include increasing age and male gender [18-19]. On the contrary, HIV-positive stroke patients are usually younger than non-HIV stroke patients. This is because younger patients are more predisposed to HIV infection, or it indicates that HIV can cause stroke independent of other vascular risk factors. The median age of HIV-positive stroke patients in developed countries like the USA is between 42 and 48 years. In developing countries like South Africa and Malawi, the median age is between 33 and 39 years [20-22]. The reason behind this variation can be the use of antiretroviral therapy in developed countries, which delays the onset of stroke in HIV patients, leading to increased median age in these countries.

HTN increases the risk of a stroke three times more than that in normotensive individuals [13]. HTN was noted in 71 (54.62%) patients in our study. Therefore, like other South Asian and Western studies, HTN was common in our study [23-24]. Patients with DM have a four-fold increased risk of stroke [5]. When compared, the frequency of DM was found to be higher in our population (18%-42.5%) than in the Western population (10%-26%) [25]. Smoking increases the risk of stroke by 1.5 to 2.9 times more than that in nonsmokers. This risk reverses back to normal after five to 10 years of smoking cessation [13]. The pattern of smoking/tobacco chewing is similar locally and in the West [26]. Dyslipidemia is undoubtedly related to the severity of carotid atherosclerosis. Despite this fact, the association between serum cholesterol and stroke risk remains a mystery [24]. Locally, dyslipidemia is estimated to have a frequency of 15.4%-32%, whereas its frequency is estimated at 22%-29% in the West [26].

In a clinical series, a large proportion (4%-34%) of HIV patients had cerebral ischemic lesions at autopsy. However, only 1%-5% of patients with HIV developed stroke when alive. Therefore, the association of cerebral autopsy lesions with clinical manifestations before death was weak [27-28]. In the last nine years, the admission of HIV-positive stroke patients has increased by 43% in the US [29]. Despite the apparent association of HIV and stroke, little attention is being paid to find out the degree of stroke risk in HIV patients and its pathogenesis [30].

## Conclusions

HIV is fairly frequent among stroke patients, especially those who are young, males, have dyslipidemia, belong to a low social-economic class, or have a sexual history indicative of a high risk of sexually transmitted diseases. Therefore, such patients should be tested for HIV. The pathogenesis of stroke in HIV patients and the role of antiretroviral therapy in the treatment and prevention of stroke due to HIV is still controversial. Therefore, further studies are needed so that a better approach can be devised to prevent and treat stroke in HIV patients.
